# Surgeon-Administered Ultrasound-Guided Regional Anesthesia in Fixation of Distal Fibula Fracture

**DOI:** 10.1155/2024/5534624

**Published:** 2024-11-08

**Authors:** Chun Lok Chow, Chun Man Ma, Tun Hing Lui

**Affiliations:** Department of Orthopaedics and Traumatology, North District Hospital, 9 Po Kin Road, Sheung Shui, New Territories, Hong Kong

**Keywords:** distal fibula fracture, fracture fixation, popliteal block, regional anesthesia, sciatic nerve block

## Abstract

Distal fibula fracture is a common injury of the lower limb. Significantly displaced distal fibula fracture is treated with surgical fixation under general, spinal, or regional anesthesia. We present a case of displaced distal fibula fracture with both the regional anesthesia and operation performed by the same attending orthopedic surgeon. The patient underwent successful ultrasound-guided regional anesthesia as well as open reduction and internal fixation. This case report highlights the technical detail for ultrasound-guided regional anesthesia and surgical fixation by surgeon.

## 1. Introduction

Distal fibula fractures are one of the commonest fractures of the lower limb [1]. Its occurrence together with other bony structure in ankle made up an annual incidence of 184 per 100,000 [2]. Distal fibula is classified according to the Weber classification basing on its anatomical level of injury [3]. For significantly displaced fracture distal fibula at level of syndesmosis, surgical management is usually recommended [4]. Neglected displaced fracture distal fibula with or without syndesmosis instability is associated with devastating complications affecting future function [5].

Surgical fixation of distal fibula fracture may require general, spinal, or regional anesthesia which are usually routinely performed by anesthetist. There are limited literatures which described distal fibula fixation performed under regional anesthesia conducted by orthopedic surgeons themselves. However, due to the shortage of anesthetist manpower in our center, we carried out the “all-surgeon-led surgery” in which selected orthopedic operations were performed under regional anesthesia administered by the orthopedic surgeons [6, 7].

In this report, we present a successful management of a patient suffering from unilateral Weber B fracture distal fibula with syndesmosis instability. Patient underwent open reduction and internal fixation under ultrasound-guided regional anesthesia performed by the attending orthopedic surgeon.

## 2. Case Presentation

**Figure 1:**
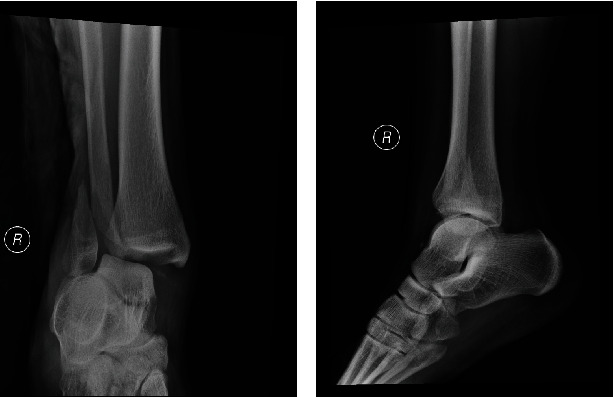
(a, b) X-ray of the right ankle AP and lateral view of the patient.

A 16-year-old boy with good past health sustained an inversion injury to right ankle during a basketball game. He was admitted to our center on the same day with complaints of pain over distal lateral ankle and failure to bear weight. On physical examination, there was bruises and swelling over his right ankle. There were no open wounds. The distal sensation, distal pulses, and capillary refill were normal. There were no signs suggestive of compartment syndrome. Radiography showed Weber B fracture of the distal fibula with talar subluxation and increased medial clear space (Figures 1(a) and 1(b)). In view of the significant fracture displacement and talar subluxation over ankle joint, close reduction under conscious sedation was performed and the fracture was immobilized with a short leg slab temporarily.

The soft tissue was allowed to rest with elevation and ice therapy. Nine days later, the swelling had resolved with positive wrinkle sign. Operation was scheduled with ultrasound-guided regional anesthesia conducted by case surgeon who would also subsequently perform the operation. The surgical plan was open reduction and internal fixation with plating to restore the anatomical alignment and stable fixation. Intraoperatively, the stability of syndesmosis would be assessed by a dynamic test, and a syndesmosis screw would be inserted if indicated.

### 2.1. Anesthetic Technique

Before the operation, the patient was fasted for 8 hours. Throughout the procedure, we ensured continuous monitoring of patient's blood pressure, pulse, SpO2, and cardiac rhythm.

**Figure 2:**
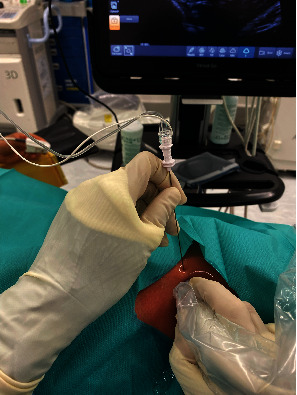
Insertion of needle under ultrasonic guidance.

**Figure 3:**
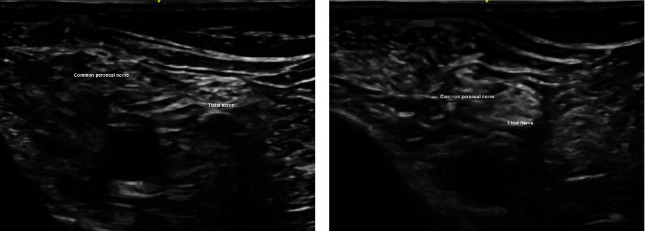
(a, b) Common peroneal nerve and tibial nerve converged as ultrasound probe, moving from caudal to cranial direction.

**Figure 4:**
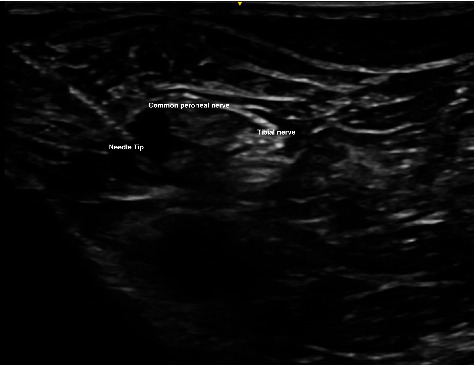
Needle positioned just underneath Vloka's sheath with infiltration of anesthetic agent.

**Figure 5:**
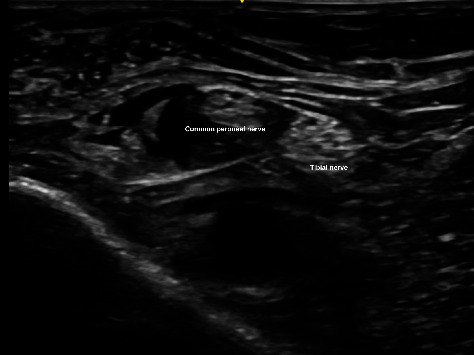
Common peroneal and tibial nerve separated by anesthetic agent.

We adopted the technique of ultrasound-guided popliteal sciatic nerve block to provide the regional anesthesia. The patient was positioned in a lateral position with the knee in a slightly flexed manner. Ultrasound screening was performed with the probe being placed over the popliteal fossa. Anatomical structures, namely, the common peroneal nerve, tibial nerve, bicep femoris, and popliteal vessels, were identified. The ultrasound probe was moved proximally to just above the popliteal fossa where the common peroneal and tibial nerves just got diverging. Twenty milliliters of mixture was prepared with 10 mL 2% lignocaine with 1:200,000 adrenaline and 10 mL 0.5% levobupivacaine. We used the mixture of anesthetic solution in order to allow faster onset of anesthesia contributed by lignocaine while allowing longer duration of effect contributed by levobupivacaine [8]. The main reason of including adrenaline in the mixture was to provide local vasoconstrictive effect which therefore reduced the absorption of anesthetic agent into systemic circulation to avoid systemic toxicity [9]. The volume of mixture used was tailored according to the patient's body weight (70 kg); in total, 20 mL of the mixture was used. After sterilization and skin preparation, a 100-mm 21 gauge insulated stimulating needle was placed in an in-plane manner from lateral to medial aiming at the Vloka's sheath which envelops the common peroneal and tibial nerve. Anesthetic mixture was injected, and a successful distribution of fluid was confirmed by separation of common peroneal and tibial nerves by the mixture. Further ultrasound scan distal confirmed spreading of anesthetic mixture surrounding the common peroneal and tibial nerves, respectively (Figures 2, 3(a), 3(b), 4, and 5).

### 2.2. Surgical Technique

**Figure 6:**
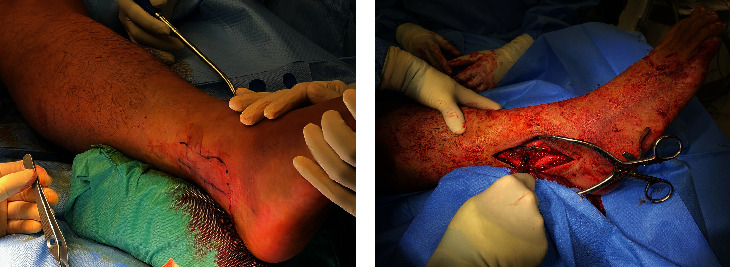
(a) Confirmation of successful induction of anesthesia with sharp needle pin prick. (b) Open reduction and internal fixation of distal fibula fracture.

**Figure 7:**
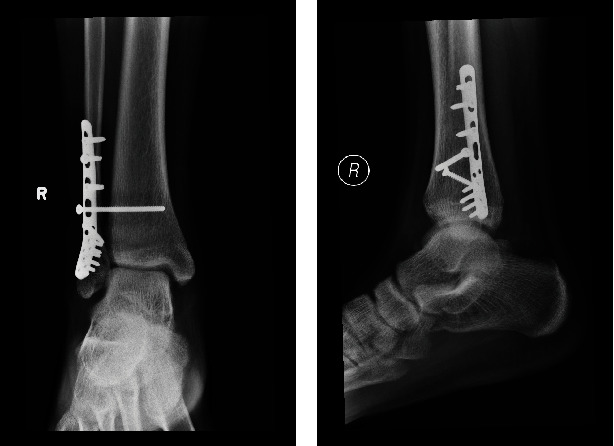
(a) X-ray of the right ankle in anterior-posterior view after operation. (b) X-ray of the right ankle in lateral view after operation.

Surgery was performed by the same surgeon who was responsible for the previous regional anesthesia. We adopted the posterolateral approach for the fixation of distal fibula. Patient was put into floppy lateral position with no tourniquet being applied. Incision was made over posterior border of distal fibula. Layered dissection with meticulous hemostasis was done till fracture seen. Fracture hematoma was cleared, and reduction was performed under direct visualization and fluoroscopy guidance. Fracture was stabilized with Synthes 2.7/3.5 distal fibula anatomical locking plate (Figures 6(a) and 6(b)). Syndesmosis stability was checked with dynamic tests intraoperatively including stress tests at coronal, sagittal, and rotation planes, which was positive. Tricortical syndesmosis screw fixation was performed across fibula and tibia, at 3 cm above tibial plafond. Surgical wound was then closed in layers, and no drain was placed. Throughout the procedure, the patient enjoyed a pain-free environment. The total operative time was 75 min, and the blood loss resulting from the operation was 20 mL. The X-ray of patient's injured ankle after operation showed anatomical reduction of fracture with restoration of the talar position (Figures 7(a) and 7(b)). The patient was discharged 4 days after the operation.

### 2.3. Rehabilitation

**Figure 8:**
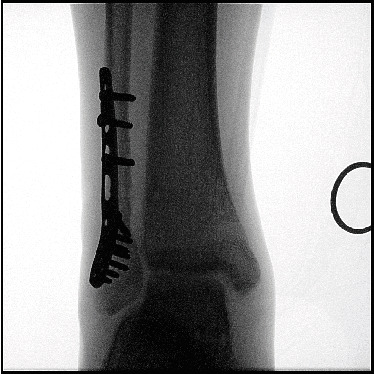
Fluoroscopy image of patient's right ankle after removal of syndesmosis screw.

The patient was allowed to have active free-range mobilization and nonweight-bearing walking for 6 weeks after rehabilitation. He was instructed to have partial weight-bearing walking for another 6 weeks before returning to full weight-bearing walking. Syndesmosis screw removal was performed 6 weeks after the initial operation under local anesthesia (Figure 8).

## 3. Result

The patient experienced complete loss of temperate sensation to cold at 8 min and complete loss of pain sensation to sharp pin prick at 19 min after administration of anesthesia.

Throughout the operation, we achieved a complete painless environment with *VAS* = 0 for the distal fibula surgical fixation. Postoperatively, we prescribed oral Panadol and tramadol as standard analgesic regimen. Pain control 6 h after operation was satisfactory with *VAS* = 2. Patient was started with nonweight-bearing walking exercise immediately after the operation. He was discharged from our hospital 4 days after the initial operation.

## 4. Discussion

Regional anesthesia is a well-established mode of analgesia for surgical patients for intraoperative pain control [10]. The safety and successful rate were further enhanced by the usage of ultrasound guidance during the procedure [11]. Different studies showed that regional anesthesia was associated with faster postoperative recovery, reduced hospital length of stay, and improved cardiopulmonary function [12]. One of the possible drawbacks of regional anesthesia for traumatic patients is the expected paresthesia after the procedure which may lower the vigilance to possible complications, for example, neurovascular injury and compartment syndrome [13]. We suggested a continuous routine postoperative monitoring of neurovascular status for a least 24 h after the operation.

It is the first ever report of orthopedic surgeon–performed popliteal sciatic nerve block in Hong Kong. In our locality, regional anesthesia is a procedure routinely carried out by anesthetist. However, due to the manpower shortage of anesthetist in our center, we started the service of orthopedic surgeon self-performed ultrasound-guided regional anesthesia for our trauma service. Our surgeons after underwent training started to performed ultrasound-guided regional anesthesia for patients with the upper limb or lower limb fracture.

Regional anesthesia, while first described, was a procedure performed by surgeon. In 1920, Dr. Cushing performed the first regional anesthesia by applying cocaine to nerve trunk during amputation. Due to the later development of the specialty of anesthesiology, regional block progressively became a procedure routinely performed by anesthetists. From our center experience, regional anesthesia performed by surgeon can provide the satisfactory level of anesthesia and safety throughout the operation.

## 5. Conclusion

According to our experience, regional anesthesia administered by orthopedic surgeons could provide painless environment for open reduction and internal fixation of distal fibula fracture.

The same principle may be applied to other traumatic or elective orthopedic conditions. But further studies need to be continued for adequate power for formal analysis of feasibility.

## Data Availability

The data used to support the findings in this study are included within the article.
